# Allergic Reactions Including Anaphylaxis After Receipt of the First Dose of Pfizer-BioNTech COVID-19 Vaccine — United States, December 14–23, 2020

**DOI:** 10.15585/mmwr.mm7002e1

**Published:** 2021-01-15

**Authors:** CDC COVID-19 Response Team, Food and Drug Administration

As of January 3, 2021, a total of 20,346,372 cases of coronavirus disease 2019 (COVID-19) and 349,246 associated deaths have been reported in the United States. Long-term sequalae of COVID-19 over the course of a lifetime currently are unknown; however, persistent symptoms and serious complications are being reported among COVID-19 survivors, including persons who initially experience a mild acute illness.* On December 11, 2020, the Food and Drug Administration (FDA) issued an Emergency Use Authorization (EUA) for Pfizer-BioNTech COVID-19 vaccine to prevent COVID-19, administered as 2 doses separated by 21 days. On December 12, 2020, the Advisory Committee on Immunization Practices (ACIP) issued an interim recommendation for use of Pfizer-BioNTech COVID-19 vaccine (*1*); initial doses were recommended for health care personnel and long-term care facility residents (*2*). As of December 23, 2020, a reported 1,893,360 first doses of Pfizer-BioNTech COVID-19 vaccine had been administered in the United States, and reports of 4,393 (0.2%) adverse events after receipt of Pfizer BioNTech COVID-19 vaccine had been submitted to the Vaccine Adverse Event Reporting System (VAERS). Among these, 175 case reports were identified for further review as possible cases of severe allergic reaction, including anaphylaxis. Anaphylaxis is a life-threatening allergic reaction that does occur rarely after vaccination, with onset typically within minutes to hours (*3*). Twenty-one cases were determined to be anaphylaxis (a rate of 11.1 per million doses administered), including 17 in persons with a documented history of allergies or allergic reactions, seven of whom had a history of anaphylaxis. The median interval from vaccine receipt to symptom onset was 13 minutes (range = 2–150 minutes). Among 20 persons with follow-up information available, all had recovered or been discharged home. Of the remaining case reports that were determined not to be anaphylaxis, 86 were judged to be nonanaphylaxis allergic reactions, and 61 were considered nonallergic adverse events. Seven case reports were still under investigation. This report summarizes the clinical and epidemiologic characteristics of case reports of allergic reactions, including anaphylaxis and nonanaphylaxis allergic reactions, after receipt of the first dose of Pfizer-BioNTech COVID-19 vaccine during December 14–23, 2020, in the United States. CDC has issued updated interim clinical considerations for use of mRNA COVID-19 vaccines currently authorized in the United States (*4*) and interim considerations for preparing for the potential management of anaphylaxis (*5*). In addition to screening for contraindications and precautions before administering COVID-19 vaccines, vaccine locations should have the necessary supplies available to manage anaphylaxis, should implement postvaccination observation periods, and should immediately treat persons experiencing anaphylaxis signs and symptoms with intramuscular injection of epinephrine (*4*,*5*).

CDC and FDA received notification of suspected anaphylaxis cases through multiple channels, including direct outreach by health care providers and public health officials and reports to VAERS, the national passive surveillance (spontaneous reporting) system for adverse events after immunization, which is jointly operated by CDC and FDA (*6*). All notifications of suspected anaphylaxis that came to the attention of CDC or FDA were also captured in VAERS. CDC physicians screened VAERS reports describing suspected severe allergic reactions and anaphylaxis and applied Brighton Collaboration case definition criteria (*7*), which use combinations of symptoms to define levels of diagnostic certainty to identify cases with sufficient evidence to warrant further assessment for anaphylaxis. Brighton level 1 represents the highest level of diagnostic certainty that a reported case is indeed a case of anaphylaxis; levels 2 and 3 represent successively lower levels of diagnostic certainty. Level 4 is a case reported as anaphylaxis but which does not meet the Brighton Collaboration case definition. Level 5 is a case that was neither reported as anaphylaxis nor meets the case definition. Reports with sufficient evidence to suggest anaphylaxis were followed up by direct outreach, including telephoning contacts listed in the VAERS report to gather additional clinical details (e.g., health care facilities and treating health care providers, and, in some cases, vaccine recipients) and collecting medical records. Physician reviewers also used their clinical judgment to categorize reports that were considered not anaphylaxis as nonanaphylaxis allergic reactions or nonallergic adverse events. Nonallergic adverse events, mostly vasovagal or anxiety-related, were excluded from the analysis. Anaphylaxis and nonanaphylaxis allergic reaction cases with symptom onset occurring later than the day after vaccination (i.e., outside of the 0–1-day risk window) were also excluded because of the difficulty in clearly attributing allergic reactions with onset later than this to vaccination.^†^ CDC and FDA conducted joint review sessions to discuss and adjudicate cases. Because the FDA EUA for the Moderna COVID-19 vaccine was received 1 week later than that for the Pfizer-BioNTech vaccine (i.e., on December 18, 2020), and the Moderna vaccine was only available beginning December 21, this report focuses on the Pfizer-BioNTech COVID-19 vaccine. An assessment of adverse events reported after receipt of the Moderna COVID-19 vaccine will be forthcoming.

During December 14–23, 2020, after administration of 1,893,360 first doses of Pfizer-BioNTech COVID-19 vaccine (1,177,527 doses in females, 648,327 doses in males, and 67,506 doses missing sex), reports of 4,393 (0.2%) adverse events after receipt of the vaccine had been submitted to VAERS. Among these, 175 case reports were identified for further review as possible cases of severe allergic reaction, including anaphylaxis, based on descriptions of signs and symptoms; 21 of these reports met the Brighton Collaboration case definition criteria for anaphylaxis, corresponding to an initial estimated rate of 11.1 cases per million doses administered. All reports were Brighton levels 1 or 2 (Table 1). The median age of persons with anaphylaxis was 40 years (range = 27–60 years), and 19 (90%) cases occurred in females. The median interval from vaccine receipt to symptom onset was 13 minutes (range = 2–150 minutes); 15 (71%) patients had onset within 15 minutes, three (14%) within 15 to 30 minutes, and three (14%) after 30 minutes (Figure). In 19 of 21 (90%) reports, patients were treated with epinephrine as part of therapy; one patient received subcutaneous epinephrine and the remaining 18 were confirmed or presumed to have received intramuscular epinephrine based on the report. Four (19%) patients were hospitalized (including three in intensive care), and 17 (81%) were treated in an emergency department; 20 (95%) are known to have been discharged home or had recovered at the time of report to VAERS. No deaths from anaphylaxis were reported after receipt of Pfizer-BioNTech COVID-19 vaccine. Seventeen (81%) of 21 patients with anaphylaxis had a documented history of allergies or allergic reactions, including to drugs or medical products, foods, and insect stings; seven (33%) patients had experienced an episode of anaphylaxis in the past, including one after receipt of a rabies vaccine and another after receipt of an influenza A(H1N1) vaccine (Table 2). No geographic clustering of anaphylaxis cases was observed, and the cases occurred after receipt of doses from multiple vaccine lots. At the time of this report, investigators have been unable to obtain sufficient information to confirm or rule out anaphylaxis in seven cases despite follow-up efforts; these cases remain under investigation.

**TABLE 1:** Characteristics of reported cases of anaphylaxis (n = 21) after receipt of Pfizer-BioNTech COVID-19 vaccine — Vaccine Adverse Events Reporting System (VAERS), United States, December 14–23, 2020

Age (yrs)	Sex	Past history		Onset after receipt (mins)	Signs and symptoms	Treatment setting^†^	Epi received	Brighton level^§^	Outcome or disposition^¶^
		Allergies or allergic reactions*	Anaphylaxis						
27	F	Tropical fruit	No	2	Diffuse erythematous rash, sensation of throat closure	ED	Yes	2	Recovered at time of report
35	M	No	No	5	Diffuse erythematous rash, swollen tongue	ED	Yes	1	Discharged home
55	F	Rabies vaccine	Yes, rabies vaccine	5	Generalized urticaria, wheezing	Inpatient	Yes	1	Discharged home
52	F	Sulfa drugs	Yes, sulfa drugs	7	Wheezing, stridor, nausea	Inpatient	Yes	1	Discharged home
30	F	Bee sting	No	8	Generalized urticaria, wheezing	Inpatient	Yes	1	Recovered at time of report
32	F	No	No	10	Diffuse erythematous rash, difficulty breathing	Inpatient	Yes	2	Discharged home
60	F	Eggs, milk, sulfa drugs, jellyfish sting	Yes, jellyfish sting	10	Diffuse erythematous rash, hoarseness	ED	Yes	2	Recovered at time of report
29	F	Shellfish, eggs	No	10	Generalized urticaria, swollen lips and tongue	ED	Yes	1	Discharged home
52	F	Metoprolol, clarithromycin	No	10	Generalized urticaria, stridor, wheezing	ED	Yes	1	Recovered at time of report
49	F	Iodinated contrast media	No	13	Generalized urticaria, swollen throat	ED	Yes	1	Recovered at time of report
36	F	No	No	13	Generalized urticaria, nausea	ED	Yes	2	Not specified
40	F	Sulfa drugs, walnuts	Yes, walnuts	14	Generalized urticaria, nausea	ED	Yes	2	Discharged home
33	F	Wasp sting	No	15	Diffuse erythematous rash, swollen lip	ED	Yes	1	Recovered at time of report
41	F	Prochlorperazine	Yes, prochlorperazine	15	Diffuse erythematous rash, persistent dry cough	ED	No	2	Discharged home
57	F	Penicillin, azithromycin	Yes, unspecified	15	Diffuse pruritic rash, hoarseness	ED	Yes	2	Recovered at time of report
45	M	No	No	23	Generalized urticaria, swollen airway	ED	Yes	2	Discharged home
46	F	Hydrocodone, nuts	No	25	Diffuse erythematous rash, difficulty swallowing	ED	Yes	2	Discharged home
30	F	Cats, dogs	No	30	Generalized pruritis, wheezing	ED	No	2	Discharged home
44	F	Influenza A(H1N1) vaccine	Yes, influenza A(H1N1) vaccine	34	Generalized urticaria, swollen lips	ED	Yes	1	Discharged home
29	F	Sulfa drugs	No	54	Generalized urticaria, persistent cough	ED	Yes	2	Recovered at time of report
29	F	Steroids	No	150	Diffuse pruritic rash, swollen lip	ED	Yes	1	Discharged home

**Abbreviations:** COVID-19 = coronavirus disease 2019; ED = emergency department; epi = epinephrine; F = female; M = male.

* As documented in the VAERS report or medical records, or through confirmation with the treating health care provider or the patients themselves.

^†^ Inpatient = inpatient hospitalization.

^§^ The Brighton Collaboration case definition uses combinations of symptoms to define levels of diagnostic certainty. Brighton Level 1 represents the highest level of diagnostic certainty that a reported case is indeed a case of anaphylaxis; Levels 2 and 3 are successively lower levels of diagnostic certainty. Level 4 is a case reported as anaphylaxis but that does not meet the Brighton Collaboration case definition. Level 5 is a case that was neither reported as anaphylaxis nor meets the case definition (https://doi.org/10.1016/j.vaccine.2007.02.064).

^¶^ As documented in the description of the adverse event in the VAERS report in Box 18 or as document in recovery status in Box 20.

**FIGURE:**
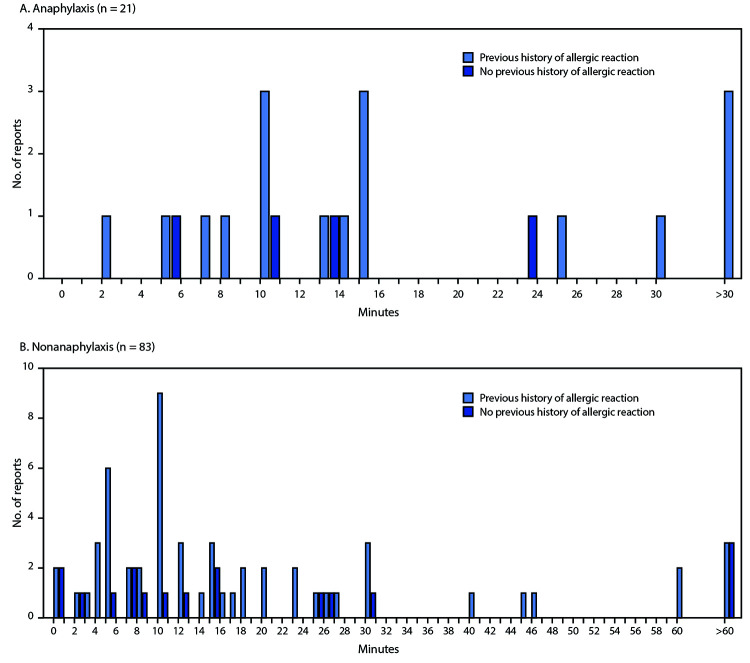
Interval (minutes) from vaccine receipt to onset of anaphylaxis (A)* and nonanaphylaxis allergic reactions (B)^†^ after receipt of Pfizer-BioNTech COVID-19 vaccine — Vaccine Adverse Events Reporting System, United States, December 14–23, 2020 **Abbreviation:** COVID-19 = coronavirus disease 2019. * The interval from vaccine receipt to symptom onset was >30 minutes for three anaphylaxis cases (34, 54, and 150 minutes). ^†^ The interval from vaccine receipt to symptom onset was >60 minutes for three nonanaphylaxis patients who had a documented history of allergies or allergic reactions at 90, 96, and 180 minutes and for three who did not have a documented history of allergies or allergic reactions (105 minutes, 137 minutes, and 20 hours). Interval from vaccine receipt to symptom onset was missing for four patients with a history of allergies or allergic reactions and for seven without such history. Three cases of nonanaphylaxis allergic reactions with symptom onset occurring later than the day after vaccination (i.e., outside of the 0–1-day risk window) were excluded from the final analysis.

**TABLE 2:** Characteristics of patients with report of anaphylaxis and nonanaphylaxis allergic reactions after receipt of Pfizer-BioNTech COVID-19 vaccine — Vaccine Adverse Events Reporting System (VAERS), United States, December 14–23, 2020

Characteristic	Type of reported reaction, no. (%)	
	Anaphylaxis (n = 21)	Nonanaphylaxis allergic reactions (n = 83)*
Median age, yrs (range)	40 (27–60)	43 (18–65)
Female	19 (90)	75 (90)
Mins to symptom onset, median (range)	13 (2–150)	12 (<1–1,200 [20 hrs])
Symptom onset ≤15 mins	15 (71)	44 (61)^†^
Symptom onset ≤30 mins	18 (86)	61 (85)^†^
Documented history of allergies or allergic reactions	17 (81)^§^	56 (67)

**Abbreviation:** COVID-19 = coronavirus disease 2019.

* Three of the initial 86 nonanaphylaxis allergic reaction reports were excluded from the final analysis because symptom onset occurred later than the day after vaccination (i.e., outside of the 0–1-day risk window).

^†^ Eleven reports were missing information on time of symptom onset; percentage calculated among 72 patients.

^§^ Seven anaphylaxis patients reported a history of a previous anaphylaxis episode, including one after receipt of rabies vaccine and one after receipt of influenza A(H1N1) vaccine.

During the same period, VAERS identified 83 cases of nonanaphylaxis allergic reaction after Pfizer-BioNTech COVID-19 vaccination with symptom onset within the 0–1-day risk window, 72 (87%) of which were classified as nonserious.^§^ Commonly reported symptoms included pruritus, rash, itchy and scratchy sensations in the throat, and mild respiratory symptoms. The median patient age was 43 years (range = 18–65 years), and 75 (90%) reported reactions occurred in women. The median interval from vaccine receipt to symptom onset was 12 minutes (range = <1 minute–20 hours); in 61 (85%) cases, onset occurred within 30 minutes, in 11 cases, onset occurred after 30 minutes, and for 11 cases, time of onset was missing. For 56 (67%) case reports, a past history of allergies or allergic reactions was documented (Table 2) (Figure).

## Discussion

Early safety monitoring of the Pfizer-BioNTech COVID-19 vaccine has detected 21 cases of anaphylaxis after reported administration of 1,893,360 first doses of Pfizer-BioNTech COVID-19 vaccine (11.1 cases per million vaccine doses administered) as well as cases of less severe nonanaphylaxis allergic reactions, based on U.S. data for December 14–23, 2020. Most (86%) anaphylaxis cases had symptom onset within 30 minutes of vaccination, and most persons with anaphylaxis (81%) had a history of allergies or allergic reactions, including some with previous anaphylaxis events; up to 30% of persons in the general population might have some type of allergy or history of allergic reactions.^¶^ Most (90%) reported anaphylaxis cases after receipt of Pfizer-BioNTech COVID-19 vaccine occurred in women, although 64% of the vaccine doses administered with sex of recipient recorded were given in women. Whereas a female predominance has been previously observed in a review of immediate hypersensitivity reports to VAERS after influenza A(H1N1) vaccine (*8*), the current finding could be impacted by the observation that more women than men had received a first dose of Pfizer-BioNTech COVID-19 vaccine during the analytic period. Anaphylaxis is potentially life-threatening and requires immediate treatment (*5*). Based on early safety monitoring, anaphylaxis after the Pfizer-BioNTech COVID-19 vaccine appears to be a rare event; however, comparisons of anaphylaxis risk with that associated with non-COVID-19 vaccines are constrained at this time by the limited data available this early in the COVID-19 vaccination program. CDC and FDA will continue enhanced monitoring for anaphylaxis among recipients of COVID-19 vaccines.

The findings in this report are subject to at least four limitations. First, the anaphylaxis and nonanaphylaxis allergic reaction case reports were gathered through passive surveillance based on spontaneous reports to VAERS. Spontaneous reporting is subject to reporting biases (including underreporting); however, the reporting efficiency to VAERS for clinically severe adverse events is believed to be high (*9*). A second potential source of bias arises from stimulated reporting related to increased public and health care provider awareness of a potential safety concern. Thus, it is possible that intense media attention around the national COVID-19 vaccination program and heightened awareness of reports of anaphylaxis have affected vaccine recipient and health care provider behavior and practices, including elevated concern and anxiety, higher index of suspicion for anaphylaxis, and lower threshold for early treatment of suspected cases, thereby resulting in an increase in diagnosis of suspected anaphylaxis and corresponding stimulated above-baseline reporting to VAERS. Third, it is possible that data lags and incomplete reporting of vaccine doses administered might underestimate the denominator (doses administered) relative to the numerator (anaphylaxis cases). If anaphylaxis cases after receipt of COVID-19 vaccine are identified and reported faster than vaccine doses administered are reported, the anaphylaxis rate associated with vaccination might be overestimated. Finally, the focus on the Pfizer-BioNTech COVID-19 vaccine is a function of the timing of product availability and doses administered. Data on the Moderna vaccine, which became available a week later, were limited. Vaccination with Moderna COVID-19 vaccine commenced on December 21, 2020, and through December 23, 2020, an estimated 224,322 first doses of the vaccine had been administered; one report that met the Brighton Collaboration case definition criteria for anaphylaxis had been submitted to VAERS.

Mortality from COVID-19 in populations at high risk is substantial (*10*), and treatment options are limited. Widespread vaccination against COVID-19 with highly effective vaccines represents an important tool in efforts to control the pandemic. CDC and FDA will continue to monitor for adverse events, including anaphylaxis, after receipt of COVID-19 vaccines and will regularly assess the benefits and risks of vaccination in the context of the evolving epidemiology of the pandemic. Continued monitoring in VAERS and additional monitoring in population-based surveillance systems, such as the CDC’s Vaccine Safety Datalink (https://www.cdc.gov/vaccinesafety/ensuringsafety/monitoring/vsd/index.html), will help to further characterize the risk for anaphylaxis after administration of COVID-19 vaccines. CDC guidance on use of mRNA COVID-19 vaccines and management of anaphylaxis is available (*4*,*5*). Specifically, vaccination locations should 1) ensure that necessary supplies are available to manage anaphylaxis, especially sufficient quantities of epinephrine in prefilled syringes or autoinjectors; 2) screen potential vaccine recipients to identify persons with contraindications and precautions (*4*); 3) implement recommended postvaccination observation periods, either 15 or 30 minutes depending on each patient’s previous history of allergic reactions; 4) ensure that health care providers can recognize the signs and symptoms of anaphylaxis early; and 5) immediately treat suspected anaphylaxis with intramuscular epinephrine; because of the acute, life-threatening nature of anaphylaxis, there are no contraindications to epinephrine administration. Patients experiencing anaphylaxis should be transported to facilities where they can receive appropriate medical care (*5*). All patients should be instructed to seek immediate medical care if they develop signs or symptoms of an allergic reaction after their observation period ends and they have left the vaccination location. Health care providers can play an important role in vaccine safety by being vigilant in recognizing and reporting adverse events after immunization to VAERS at https://vaers.hhs.gov/reportevent.html.

**Summary**

**What is already known about this topic?**

Anaphylaxis is a severe, life-threatening allergic reaction that occurs rarely after vaccination.

**What is added by this report?**

During December 14–23, 2020, monitoring by the Vaccine Adverse Event Reporting System detected 21 cases of anaphylaxis after administration of a reported 1,893,360 first doses of the Pfizer-BioNTech COVID-19 vaccine (11.1 cases per million doses); 71% of these occurred within 15 minutes of vaccination.

**What are the implications for public health practice?**

Locations administering COVID-19 vaccines should adhere to CDC guidance for use of COVID-19 vaccines, including screening recipients for contraindications and precautions, having the necessary supplies available to manage anaphylaxis, implementing the recommended postvaccination observation periods, and immediately treating suspected cases of anaphylaxis with intramuscular injection of epinephrine.

## Footnotes

* https://www.cdc.gov/coronavirus/2019-ncov/hcp/clinical-care/late-sequelae.html.

† Anaphylaxis and nonanaphylaxis allergic reaction cases with symptom onset occurring later than the day after vaccination (i.e., outside of the 0–1-day risk window) were excluded because of the difficulty in clearly attributing allergic reactions with onset outside this risk window to vaccination. Three of the initial 86 nonanaphylaxis allergic reactions were excluded from the final analysis.

§ Based on the Code of Federal Regulations, a serious adverse event is defined if one of the following is reported: death, life-threatening illness, hospitalization or prolongation of hospitalization, permanent disability, congenital anomaly, or birth defect. https://www.accessdata.fda.gov/scripts/cdrh/cfdocs/cfcfr/cfrsearch.cfm?fr=312.32.

¶ https://www.aaaai.org/about-aaaai/newsroom/allergy-statistics.
